# Integrative Analysis Identified CD38 As a Key Node That Correlates Highly with Immunophenotype, Chemoradiotherapy Resistance, And Prognosis of Head and Neck Cancer

**DOI:** 10.7150/jca.59730

**Published:** 2023-01-01

**Authors:** Zhengxi He, Chunxue Yue, Xiuwen Chen, Xin Li, Li Zhang, Shan Tan, Xia Yi, Gengqiu Luo, Yanhong Zhou

**Affiliations:** 1 Hunan Cancer Hospital and The Affiliated Cancer Hospital of Xiangya School of Medicine, Central South University Changsha, Hunan, 410013, China.; 2 NHC Key Laboratory of Carcinogenesis, Hunan Cancer Hospital and The Affiliated Cancer Hospital of Xiangya School of Medicine, Central South University, Changsha, Hunan, 410013, China.; 3 Department of Oncology, Xiangya Hospital, Central South University, Changsha, Hunan, 410008, China.; 4 Cancer Research Institute, Basic School of Medicine, Central South University, Changsha, Hunan, 410011, China.; 5 Department of Otolaryngology-Head and Neck Surgery, Shandong Provincial ENT Hospital, Jinan, Shandong, 250022, China.; 6 Teaching and Research Section of Clinical Nursing, Xiangya Hospital, Central South University, Changsha, Hunan, 410008, China.; 7 Breast Cancer Center, Department of General Surgery, Xiangya Hospital, Central South University, Changsha, Hunan, 410008, China.; 8 Changsha Medical University, Changsha, Hunan, 410219, China.; 9 Department of Pathology, Xiangya Hospital, Basic School of Medicine, Central South University, Changsha, Hunan, 410008, China.

**Keywords:** HNC, Head and neck cancer, CD38, Radioresistance, PD-1/PD-L1, Prognosis

## Abstract

Head and neck cancer (HNC) is mainly treated by surgery, radiotherapy, and adjuvant chemotherapy; however, the prognosis of some patients with HNC is poor because of radiotherapy and chemotherapy resistance. In recent years, anti‑PD‑1 monoclonal antibodies have shown certain efficacy, and a change of the tumor immune microenvironment is the main reason for the failure of HNC immunotherapy. The present study aimed to identify and verify that CD38, which is closely related to the prognosis of HNC, is a potential biological marker of radiotherapy and chemotherapy resistance and PD-L1 immunotherapy resistance via a comprehensive bioinformatic analysis in The Cancer Genome Atlas and Gene Expression Omnibus databases. According to the UALCAN database, the transcript level of *CD38* in HNC was analyzed using cluster analysis, and the expression of *CD38* mRNA in HNC was detected using the Oncomine database. The characteristics of CD38-related oncogenes were identified by gene cluster enrichment analysis in LinkedOmics. The R2 and SEER databases were used to further evaluate the prognostic significance of the *CD38* gene in HNC using receiver operating characteristic curve analysis of Kaplan-Meier (KM) survival and the clinical characteristics of the subjects. The protein-protein interaction network of the top 50 genes showing significant positive correlations with CD38 in HNC was analyzed using STRING. Finally, we used a nasopharyngeal carcinoma (NPC) cell line to verify the biological function. The results showed that the levels of *CD38* mRNA expression in patients with HNC were significantly higher than those in healthy controls. The levels of CD38 mRNA expression in patients with HNC of different ages, sexes, and races were significantly higher than those in the healthy controls. CD38 is an independent prognostic factor for HNC, and high expression of CD38 indicates poor prognosis. CD38 expression correlated positively with the markers of many kinds of immune cells, and correlated significantly with the expression of PD-L1. We found that the high expression of CD38 suggested a poor prognosis in the subgroup of tumors treated with chemotherapeutic drugs in the G1/S phase. We used HNC cell lines to verify that the high expression of CD38 promoted the proliferation of NPC cells and produced radiotherapy tolerance. Through comprehensive bioinformatics analysis, we suggested that *CD38* is a key gene involved in radiotherapy, chemotherapy, and immune drug resistance in HNC. This study provides a reliable biomarker to predict the prognosis of patients with HNC and a reference for clinical comprehensive treatment of HNC. Individualization combined with CD38 monoclonal antibodies might provide a promising treatment strategy for this fatal disease, and this comprehensive treatment might reduce the damage to normal tissue and improve the prognosis and quality of life of patients with HNC.

## Introduction

Head and neck squamous cell carcinoma (HNSC) are one of the most common tumors worldwide, ranking eighth in terms of incidence. It is estimated that there will be about 53260 new cases of oral, pharyngeal, and laryngeal cancer in the United States in 2020, accounting for about 4% of new cancer cases. During the same period, 10750 people will die of HNSC 1. More than 90% of the histological types of HNSC are squamous cell carcinoma (HNSCC) 2. Alcoholism and smoking are the most common causes of oral, hypopharyngeal, laryngeal, and oropharyngeal cancer unrelated to human papillomavirus (HPV). The entire respiratory epithelium is exposed to these possible carcinogens; therefore, patients with HNSC tumors might be at risk of developing secondary primary cancers of HNSC, in the lung, esophagus, and other sites that also have these risk factors 3-4. At present, surgery or radiotherapy is generally recommended for about 30 to 40% of patients with early stage disease (stage I or II), and radiotherapy is the first choice for nasopharyngeal carcinoma (NPC) 5. However, because about 60% of patients with HNSC are diagnosed with locally advanced stage disease, the benefit of comprehensive treatment for most patients is very limited, and their 5-year survival rate is less than 40% 6. Radiotherapy resistance and local recurrence and metastasis are observed commonly in the clinic. Therefore, identifying the key markers of radiotherapy and chemotherapy resistance is very important to improve patient prognosis.

The molecular mechanism of chemoradiotherapy resistance mediated by HNSC is not clear, and cancer cells seem to cause this phenomenon in many ways. One widely accepted concept is the role of cancer stem cells (CSCs). CSCs are defined as a group of undifferentiated cells with the ability of self-renewal, which can lead to tumor maintenance and progression 7. We previously reported that SP (side population) cells with a CSC function play a role in promoting the proliferation and metastasis of NPC by activating zeta chain of T cell receptor associated protein kinase 70 (ZAP70) to regulate the phosphatidylinositol 3-kinase (PI3K)-protein kinase B (Akt) pathway in NPC cells. Cluster of differentiation 38 (CD38) can endow cells with CSC characteristics, including promoting cell proliferation and progression by activating matrix metalloproteinase 9 (MMP9), one of the markers of epithelial-mesenchyme transition (EMT) 8. Previously, we reported that CD38 promotes cell transformation to the S phase and reduces the content of reactive oxygen species and Ca^2+^, and determined and verified that CD38 plays an important role in regulating tumor protein 53 (P53), hypoxia inducible factor-1 α (HIF-1α), and sirtuin1 (SIRT1), which are highly related to radiotherapy and chemotherapy tolerance. Taken together, these results suggested that CD38 might play a key role in the tolerance of HNSC to radiotherapy and chemotherapy.

In the present study, through integrated bioinformatic analysis, we analyzed the expression of CD38 in head and neck squamous cell carcinoma, its survival correlation, and its effect on the immune microenvironment in multiple tumor biological databases. The differences in transcription and expression were further verified in nasopharyngeal carcinoma cell lines. We also explored the effect of the *CD38* gene on the malignant biological behavior of nasopharyngeal carcinoma cells from the point of view of cell proliferation, and discussed its downstream mechanism. We confirmed that CD38 is overexpressed in NPC (in a radiation-tolerant cell line). Overexpression of CD38 in an NPC cell line could lead to radiotherapy tolerance, which might act via the PI3K-Akt pathway. It is worth noting that overexpression of* CD38* could also induce programmed cell death 1 ligand 1 (PD-L1) positivity suggesting that CD38 might participate in HNSC immunotherapy. Taken together, our data suggested that CD38 plays a key role in enhancing radiation resistance, and targeting both CD38 and PD-L1 might be a useful therapeutic strategy to treat HNSC.

## Materials and methods

### HNC Database

The HNC database (http://hncdb.cancerbio.info) integrates high-throughput gene expression data of HNC collected from the Gene Expression Omnibus (GEO) and The Cancer Genome Atlas (TCGA) databases and literature text mining in Pubmed abstracts to provide a comprehensive collection of data for HNC-related genes and drugs 9. We obtained the clinical data related to HNC from the “data” module of the HNCDB.

### GEPIA Dataset

The interactive online database GEPIA (Gene Expression Profiling Interactive Analysis) (http://gepia.cancer-pku.cn/index.html) was used to compare the mRNA expression level of *CD38* in 33 different human cancers. GEPIA is a newly developed web portal for analyzing RNA sequencing expression data from the TCGA and Genotype-Tissue Expression (GTEx) projects, including 9736 tumors and 8587 normal samples 10.

### Oncomine analysis

Oncomine (www.oncomine.org) is the biggest cancer microarray database and integrated data mining platform, which integrates the high-quality tumor tissue chip data from the literature and chip databases, including 715 gene expression datasets and 86733 cancer and normal tissues to date 11. We analyzed *CD38* mRNA levels in HNSC compared with normal samples using the Oncomine 4.5 database, which drew on a series of HNSC studies, including Ginos Head-Neck, Pyeon Multi-cancer, Toruner Head-Neck, and Cromer Head-Neck datasets 12-15. The threshold was determined as: p-value < 0.01.

### UALCAN analysis

UALCAN (http://ualcan.path.uab.edu) is a website for the online analysis and mining of the TCGA database, which is based on PERL-CGI, javascript, and css, including TCGA level 3 RNA-seq and clinical data from 31 cancer types. The portal also provides expression level, survival analysis, correlation analysis, and DNA promoter methylation data analysis. Using UALCAN, we investigated the expression of *CD38* in patients with HNSC compared with healthy individuals in subgroup analyses based on disease stages, ethnicity, gender, and age. In addition, we also used UALCAN to analyze the relationship between the expression levels of stage and CD38 in different tumors in HNSC and the survival rate of HNSC patients.

### LinkedOmics

LinkedOmics (http://www.linkedomics.org/login.php) is a publicly available online analysis website that collects data on 32 TCGA cancer types and proteomics data generated by CPTAC (Clinical Proteomics Tumor Analysis Consortium) based on mass spectrometry 16. We used the Pearson correlation test in the Linkfinder module of LinkedOmics to analyze the genes that correlated significantly with CD38 in TCGA data from 520 patients with HNSC with false discovery rate [FDR] of 0.01. The results from Linkfinder were then sent to the GESA (Gene Set Enrichment Analysis) tool from the LinkInterpreter module to perform GO (cellular component (CC), biological process (BP), and molecular function (MF)), Koto Encyclopedia of Genes and Genomes (KEGG) pathway and network analyses with an FDR < 0.05.

### R2

We used the “Kaplan Meier” module of the R2 Gene Analysis Visualization platform (https://hgserver1.amc.nl/cgi-bin/r2/main.cgi) to obtain an overall survival (OS) microarray dataset (Tumor Head Neck Squamous Cell Carcinoma-TCGA-520-rsem-tcgars) to analyze the relationship between *CD38* gene expression and OS in patients with HNSC. Using the last quartile of* CD38* expression as the cutoff, all OS data were divided into low and high groups. P < 0.05 was considered to be statistically significant.

### STRING

To construct a protein-protein interaction (PPI) network between the top 50 genes that correlated significantly and positively with* CD38* obtained from Linkedomics, we use the STRING (https://string-db.org/) database to collect, score, and integrate the input information, which was further supplemented by computational predictions. The STRING online database currently contains the largest number of organisms, reaching 5090, containing 24.6 million proteins.

### TIMER

The TIMER online database (https://cistrome.shinyapps.io/timer/) was used to systematically analyze the level of tumor infiltrating immune cells in 6 of 10897 samples of 32 TCGA tumors. We first used the “gene” module in TIMER to analyze the relationship between the expression of *CD38* in HNSC and the infiltration abundance of B cells, CD8+ T cells, CD4+ T cells, macrophages, neutrophils, and dendritic cells. We further used the "correlation" module in TIMER to analyze the relationship between specific biomarkers levels of different immune cells and *CD38* expression in HNSC. The X-axis is set to the *CD38* gene, and Y-axis is set to a specific biomarker gene (Figure 5 and 6).

### GDSC Database

GDSC (Genomics of Drug Sensitivity in Cancer) (https://www.cancerrxgene.org/) is the largest public database that provides information on tumor drug sensitivity and identifies molecular markers of drug responses, describing the responses of 1000 human cancer cell lines to more than 100 anticancer drugs so far. We summarized 21 pathways that are sensitive to 518 chemotherapeutic drugs from the GDSC database.

### Patients' specimens

The study material consisted of 15 tumor tissue samples are obtained during biopsy. The inclusion criteria were as follows: (1) histological confirmed NPC; (2) PD-L1 positive; (3) complete clinicopathological and follow-up data. The exclusion criteria were as follows: (1) histological diagnosed second primary tumor; (2) history of preoperative chemotherapy/radiation therapy. All samples are immediately sent to Pathology Department for sealing in wax block.

### Cell Culture

Human Nasopharyngeal carcinoma (NPC) cell lines (CNE2, HNE2, 6-10B, H0NE1, HK1, and C666-1) were purchased from the ATCC (Manassas, VA, United States) and maintained in our laboratory. Cell lines were maintained at 37 °C in a 5% CO_2_ atmosphere in Roswell Park Memorial Institute (RPMI) 1640 (Thermo Fisher Scientific, Waltham, MA, USA) containing 10% fetal bovine serum (Gibco Life Technologies, Grand Island, NY, USA).

### Establishment of radiation resistant cell lines

To establish radio-resistant subclones, HNE2 and CNE2 parental cells were irradiated with 2 Gy, four times, once every week for ten weeks. HNE2 and CNE2 cells were made resistant to radiation and designated as HNE2-IR or CNE2-IR.

### Colony Formation, CCK-8 Assay, and radiosensitivity assay

Cell proliferation was evaluated using colony formation and Cell Counting Kit-8 (CCK-8) assays (Beyotime Biotechnology, Shanghai, China) under radiation. NPC cells were seeded in six-well plates for different radiation doses to allow for an approximately equal number of resultant colonies, the optimal number of cells was determined to be 2000-3000 per well. The following day, cells were irradiated using a high-dose-rate Varian Clinac 23EX (Varian Medical Systems, Inc., Palo Alto, CA, USA) irradiator and cultured for 10 to 14 days to allow for colony formation. Cells were then fixed in 4% paraformaldehyde solution and stained using 0.3% crystal violet. Colonies of more than 50 cells were then counted and the survival fraction was determined. Colonies were also quantified using the ImageJ software (NIH, Bethesda, MD, USA). All treatments were performed in triplicate or higher. For the CCK-8 assay, cells were seeded in 96‑well culture plates at a density of 1,000 cells/well in 200 μl of medium. After culture at 37 °C for 6 days, we analyzed the cell density in each well every 24 h using the 7Sea-Cell Counting Kit (7Sea Biotech, Shanghai, China). Briefly, 20 μl of CCK-8 solution was added to each well and incubated for 2 h at 37 °C before the absorbance at 450 nm was measured using a Paradigm Detection System (Beckman Coulter, Brea, CA, United States).

### Western Blotting Analysis

For western blotting analysis of protein levels, all cells were lysed in radioimmunoprecipitation (RIPA) buffer (CWBio, Beijing, China). Then, 50 μg lysate was electrophoresed on 10% separation gel and transferred to a polyvinylidene fluoride (PVDF) membrane (HyClone Laboratories, Logan, UT, USA), which was blocked with 5% non-fat milk diluted in phosphate-buffered saline (PBS)-Tween20 for approximately 2 h. Primary antibody solution was then added and incubated at 4 °C for 12 h. The primary antibodies used were: Anti-phospho-phosphatidylinositol 3-kinase (PI3K) p85 alpha (Tyr607), anti-phospho-PI3 Kinase p85 beta (Ser605), anti‑phospho‑PI3K p85 (Tyr458), anti-CD38, anti-phosphatase and tensin homolog deleted on chromosome ten (PTEN), anti-NF-κb, anti-mTOR, anti-p53, and anti-PD-L1 (at dilution ratios of 1:500-1:2000 using 5% Skim milk, Affinity Biosciences (Cincinnati OH, USA) supplied the antibodies for PI3K, CD38, PTEN, and Cell Signaling Technology (Danvers, MA, USA) supplied the PD-L1 antibodies. After three washes on a shaker with PBS-Tween 20, the membranes were incubated with anti-rabbit or anti-mouse horseradish peroxidase (HRP)-conjugated secondary antibody (Santa Cruz Biotechnology, Santa Cruz, CA, USA, 1:5000 dilution) for 1 h at 37 °C). Finally, the immunoreactive protein bands were developed using the Luminata Forte western HRP substrate (Millipore, Billerica, MA, USA). Anti-GAPDH (Santa Cruz Biotechnology) levels were detected for normalization.

### Immunohistochemistry

Protein expression detected by IHC was performed on NPC pathological sections (biopsy tissue, without any treatment). We obtained formalin-fixed, paraffin-embedded NPC specimens (15 patients) from the Department of Pathology, Xiangya Hospital of Central South University and prepared tissue sections (5 µm). The specimens were immunostained using the UltraVision Quanto horseradish peroxidase detection system (Thermo Fisher Scientific). After routine deparaffinization with a series of xylene and alcohols, antigen retrieval was performed using 90% formic acid. Slides were then rinsed with distilled H2O and wash buffer. Endogenous peroxidase activity was blocked with H2O2 solution (TA-125-HP, Thermo Fisher Scientific) for 10 min prior to incubation with mouse anti-CD38 monoclonal antibody (14-0381-82, Thermo Fisher Scientific Company) at 1:100 for 60 min at room temperature. The primary antibody signal was developed with Quanto detection reagents and 3,3ʹ-diaminobenzidine chromogen as per the manufacturer's instructions. Virtual slides were produced by scanning the immunohistochemical (IHC) glass slides using the KFBIO digital pathology scanner. Digital quantitative analysis of CD38 immunoreactivity in cells was performed by an experienced pathologist in a blinded manner with K-viewer (KFBIO technology company) using a customized positive pixel count algorithm. CD38 expression was evaluated by using IHC scores. Stain intensity values are provided as a scoring system for each chromophore comprised of staining intensity and extensiveness captured the outcome: 0, negative; 1, weak; 2 moderate; 3, strong. Each field was scored independently by two pathologists.

### Statistical analysis

Differences between the groups were assessed using Student's t-test or the Fisher's exact test. Two-tailed *P* values of less than 0.05 were considered significant. All statistical analyses were performed using the GraphPad Prism 9.0 software program (GraphPad Software, Inc. La Jolla, CA, USA).

## Results

### Characteristics of Global Development Trend of HNSC

**Figure 1:**
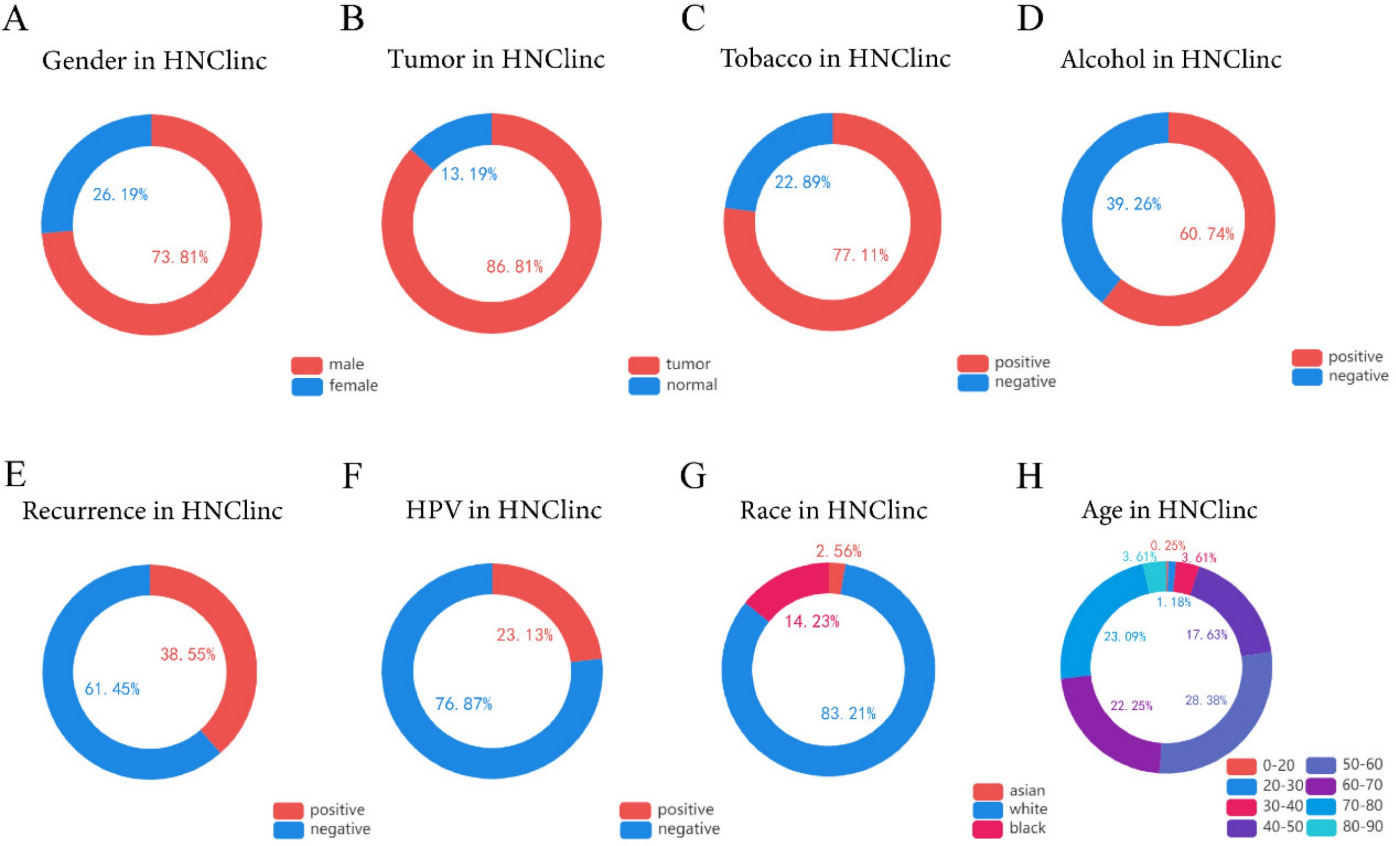
** The global clinical characteristics of HNSC. (A)** Circle showing the ratio of males to females among patients with HNSC. **(B)** Circle showing the proportion of tumor and normal tissue in biopsy samples of patients with HNSC. **(C)** Circle showing the proportion of people with smoking habits among HNSC patients. **(D)** Circle showing the proportion of people with drinking habits among HNSC patients. **(E)** Circle Chart showing the proportion of recurrence in patients with HNSC. **(F)** A circle chart showing the proportion of patients with HNSC detected to have an HPV infection. **(G)** Circle chart showing the proportions of Asians, Caucasians, and Afro-Caribbeans among the patients with HNSC. **(H)** Circle chart showing the proportion of patients with HNSC in each age range of 0-20, 20-30, 30-40, 40-50, 50-60, 60-70, 70-80, and 80-90 years old.

We used the HNC database (http://hncdb.canceromics.org/) to analyze clinical data related to the global status of HNSC (Figure 1). Through the analysis of 2403 clinical samples obtained from the NCBI GEO and TCGA databases, we found that most of the patients with HNSC were male (n=1288/1745, 73.81%), HNSC predominantly occurred in middle-aged people, and about 91.35% of the patients with HNSC were 40-80 years old at the time of onset (n = 1088/1191). Drinking (n = 362/596, 60.74%) and smoking (n = 549/712, 77.1%) habits were risk factors for HNSC. HPV is also one of the risk factors of HNSC 17-18, and viral infection might recruit immune effector cells and upregulate PD-1 and cytotoxic T-lymphocyte associated protein 4 (CTLA-4) immunosuppressive pathways. In summary, about 1/4 of the patients with HNSC were HPV-positive- (n = 133/575, 23.13%), most of them with HPV-16 infection 19; therefore, it is necessary for men to undergo HPV vaccination and routine HPV infection examination if conditions permit. The main cause of death in patients with HNSC is recurrence and metastasis. We were surprised to find that the recurrence rate of HNSC was as high as 38.55% (n = 62/166). Thus, more effective individualized treatment of HNSC is imperative.

### The relationship between CD38 and HNSC

#### *CD38* is highly expressed in HNSC

**Figure 2:**
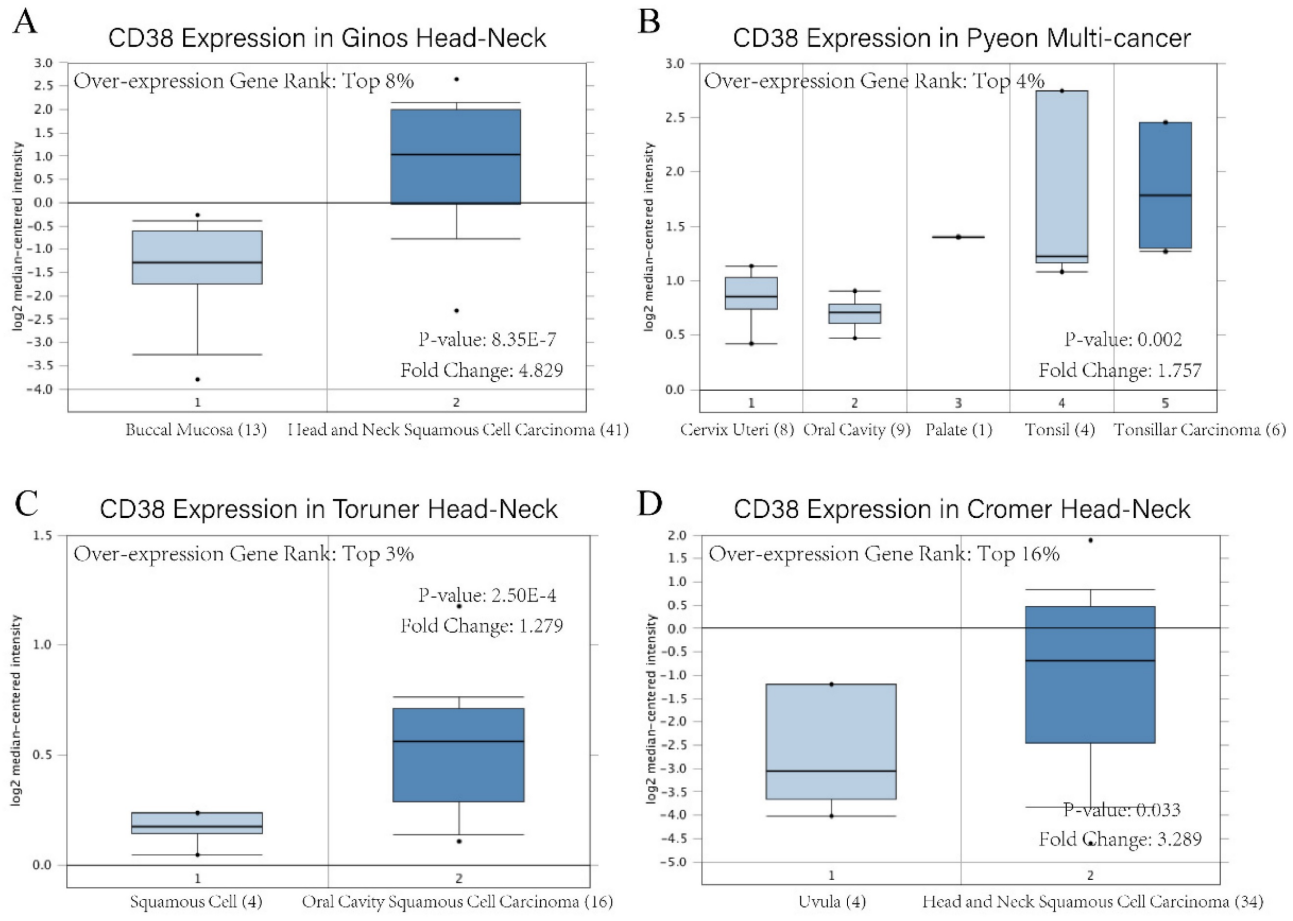
***CD38* mRNA level of in head and neck squamous cell carcinoma**. Overexpression gene rank, fold change, and related *p*-values are shown, based on Oncomine 4.5 analysis.** (A-D)** Box plot showing* CD38* mRNA expression in Ginos Head-Neck, Pyeon Multi-cancer, Toruner Head-Neck, and Cromer Head-Neck datasets, respectively.

**Figure 3:**
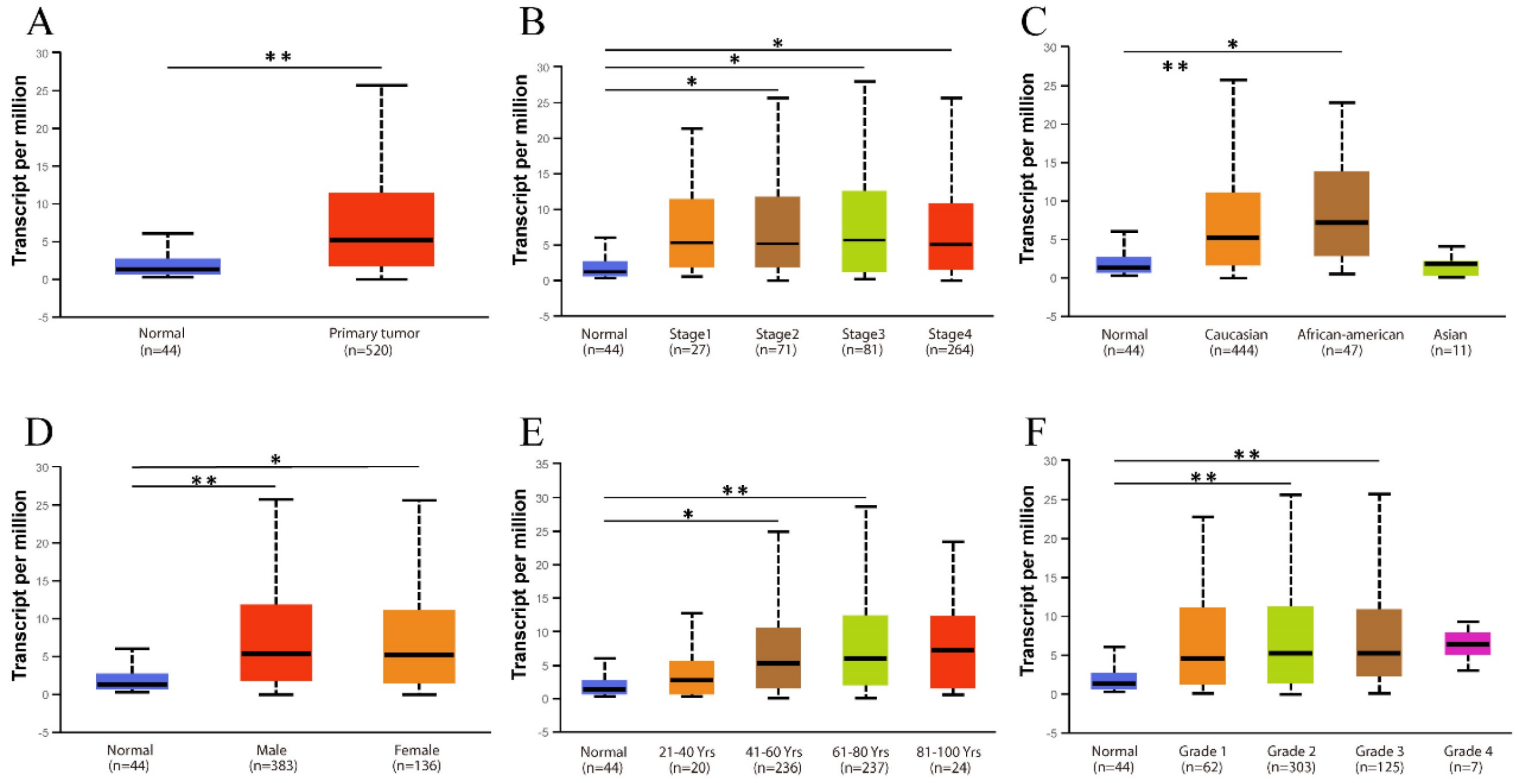
** Relationship between CD38 expression and clinical parameters in healthy subjects and patients with HNSC. (A)** Box diagram showing the relative transcript levels of *CD38* in HNSC and healthy samples. **(B)** Relative *CD38* transcription level in healthy individuals or in patient with HNSC in disease stage 1, 2, 3, or 4 shown as a box plot. **(C)** Relative *CD38* transcription level in healthy individuals of any race or in patients with HNSC of Caucasian, African-American, or Asian ethnicity, shown using a box plot. **(D)** Relative C*D38* transcription level in healthy individuals of either gender or in female or male patients with HNSC, shown as a box plot. **(E)** Relative *CD38* transcription level in healthy individuals with any age or in patients with HNSC at ages of 21-40, 41-60, 61-80, or 81-100 years, shown as a box plot. **(F)** Relative *CD38* transcription level in healthy individuals or in patients with HNSC with tumor grade 1, 2, 3, or 4, as shown in a box plot. Data are the mean ± SE. *, *P* < 0.05; **, *P* < 0.01; ***, *P* < 0.001.

The existence of oncogenes and inducing factors mean that HNSC often secretes some special proteins in the process of its occurrence and development. Researchers are able to track and quantify the expression level of these proteins and their relationship with the clinical characteristics and prognosis of tumors 20-22. We use the open Oncomine database to further detect *CD38* mRNA expression levels in HNSC. *CD38* expression in the HNSC group was significantly higher than that in the healthy control group. Ginos reported that *CD38* expression was 4.829-fold higher in HNSC compared with that in buccal mucosa tissues (p = 8.35E-7). Another study from Cromer also showed a significant increase in the *CD38* mRNA level in HNSC compared with that in uvula tissues (3.289-fold increase, p = 0.033) 12-15. In addition, in Pyeon's and Toruner's studies, although the fold-increase was less than 2, based on the mRNA level, *CD38* ranked within 5% of mRNA fold change (Figure 2). To further study the transcript levels of *CD38* in HNSC in association with a variety of clinicopathological features in the TCGA, we carried out subgroup analysis using the UALCAN database. We found that* CD38* expression in patients with HNSC was significantly higher than that in healthy subjects according to disease sorted by stage, race, sex, age, and cancer grade (Figure 3).

#### The high expression of *CD38* is associated with poor prognosis of HNSC

**Figure 4:**
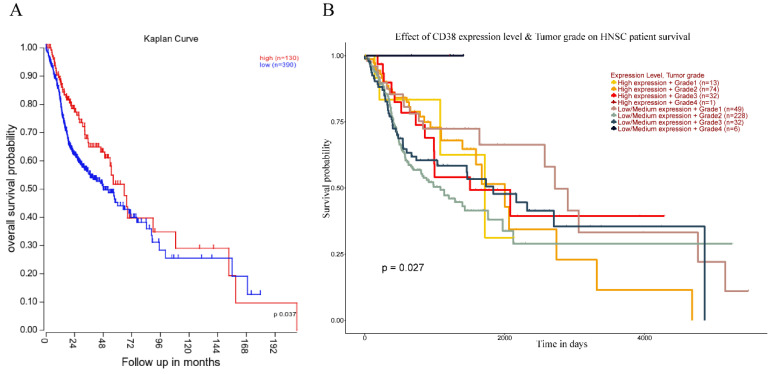
** Relationship between prognosis and CD38 expression in patients with HNSC. (A)**The relationship between the expression of CD38 and OS (overall survival) in patients with HNSC. **(B)** Relationship between the tumor grade and CD38 in different clinical tumors of HNSC and the OS in patients with HNSC.

Next, we analyzed the prognostic value of *CD38* expression in HNSC using the R2 database (Figure 4). High expression of *CD38* in HNSC was associated with poor prognosis (p = 0.037). In addition, we used the UALCAN database to verify the prognostic value of *CD38* in HNSC. There was no significant relationship between the expression of *CD38* and the staging of HNSC, suggesting *CD38* could be detected in HNSC patients with all stages of the disease. In Grade 2, the prognosis of patients with low expression of *CD38* was significantly better than that of those with high expression (p = 0.027).

### The expression of *CD38* is closely related to the immune microenvironment of HNSC

**Figure 5:**
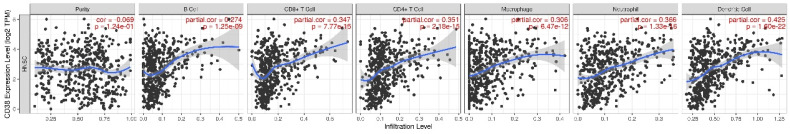
CD38 is closely related to HNSC immune microenvironment.

**Figure 6:**
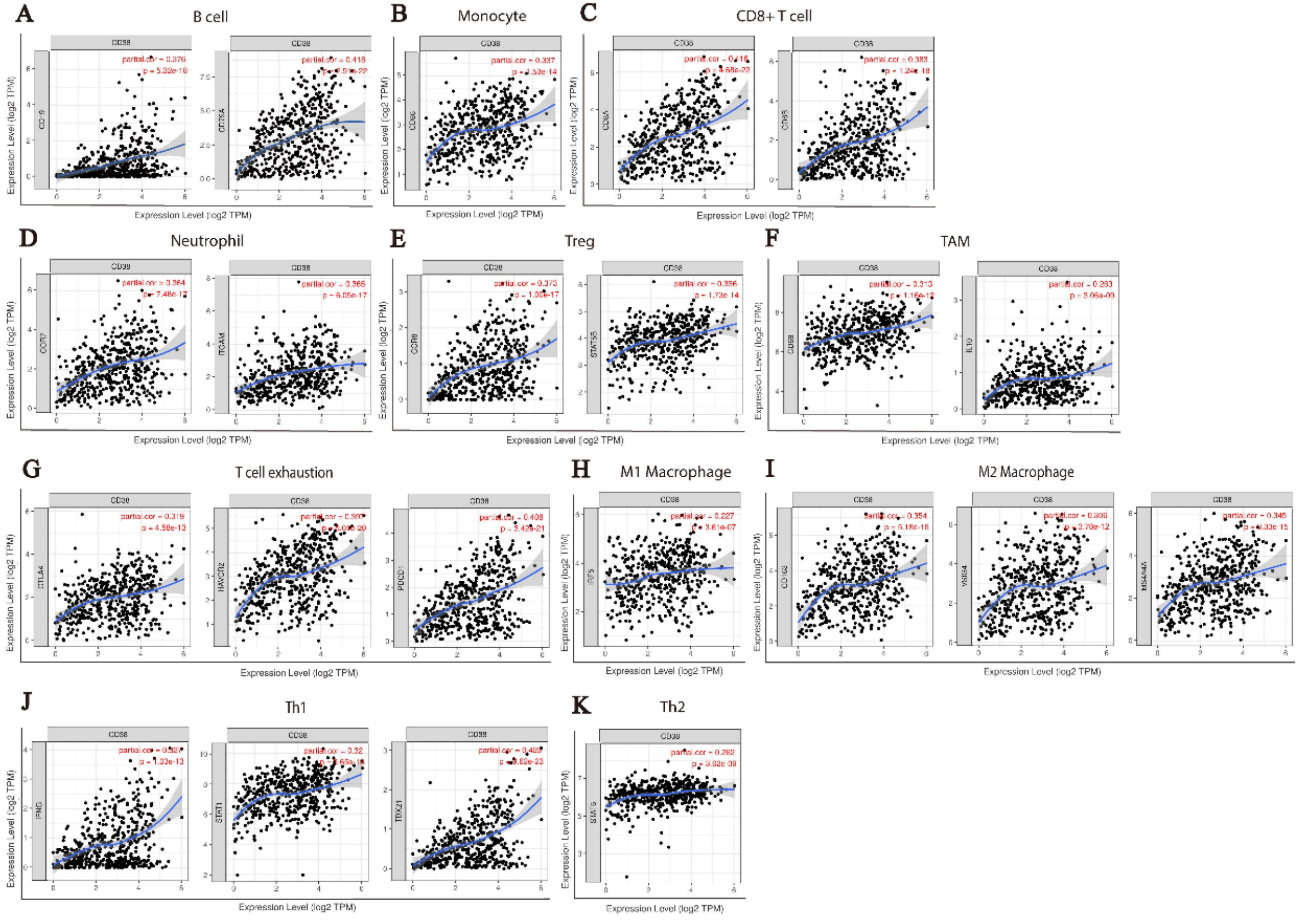
** CD38 and the number of immune cells in the HNSC microenvironment correlate positively.** The TIMER database was used to analyze the correlation between the expression of CD38 in HNSC and the expression of biomarkers in immune infiltrating cells. **(A-K)** Scatter plots representing correlation between CD38 expression and the biomarkers of (A) B cells (CD19 and CD79A); (B) Monocytes (CD86); (C) CD8+ T cells (CD8A and CD8B); (D) Neutrophils (CCR7 and ITGAM); (E) Tregs (CCR8 and STAT5B); (F) TAMs (CD68 and IL10); (G) Exhausted T cells (CTLA4, HAVCR2 and PDCD1); (H) M1 Macrophages (IRF5); (I) M2 Macrophages (CD163, MS4A4A and VSIG4); and (J) Th1 cells (IFNG, STAT1, and TBX21)) in HNSC samples. (K) Th2 cells (STAT6).

At present, the use of immune checkpoint inhibitors (ICIs) has become a popular treatment for HNSC worldwide, which brings hope of cure for patients with recurrent HNSC. However, only a small number of patients with cancer who received immune checkpoint therapy (ICT) responded, which was related to the tumor immune microenvironment (TIME) subtypes of HNSC and the presence of tumor infiltrating lymphocytes (TIL) 23. Through the classification and study of immune subtypes of HNSC, customized immunotherapy for different HNSC subsets will become a potential treatment in the future 24-26. We used TIMER to analyze the relationship between the expression of* CD38* in HNSC and the infiltration of various immune cells, as shown in Figure 5. In HNSC, the expression of *CD38* and B cells (correlation (Cor) = 0.274. P = 1.25e-09), CD8+T cells (Cor = 0.347, P = 7.77e-15), CD4+T cells (Cor = 0.351, P = 2.18e-15), macrophages (Cor = 0.306, P = 6.47e-12), neutrophils (Cor = 0.366, P = 1.33e-16), and dendritic cells (Cor = 0.425, P = 1.60e-22) showed significant positive correlations. Then, we further analyzed the correlation between *CD38* expression in HNSC and immune marker genes. As shown in Table 1 and Figures 5 and 6, the results suggested that there is a significant positive correlation between the expression of *CD38* and the markers of many kinds of immune cells in HNSC.

We further detected the expression of PD-L1 in the constructed NPC cell line overexpressing *CD38*. Interestingly, the overexpression of CD38 activated the expression of PD-L1 (Figure 10A). The close relationship between CD38 and PD-1/PD‑L1 not only occurs in HNSC, but also in other cancers. For example, Chen et al. observed upregulation of CD38 in patients with PD-1/PD-L1-resistant non-small cell lung cancer, and CD38 and adenosine pathways were involved in mediating immunosuppression in PD-1/PD-L1 therapy 27. In the future, CD38, as a new biomarker, should be used in the routine detection of HNSC. The combined use of a CD38 inhibitor and ICI in HNSC should amplify the efficacy of immunotherapy and improve the efficacy of radiotherapy and chemotherapy.

### Effect of CD38 on the tolerance of HNSC to radiotherapy and chemotherapy

**Figure 7:**
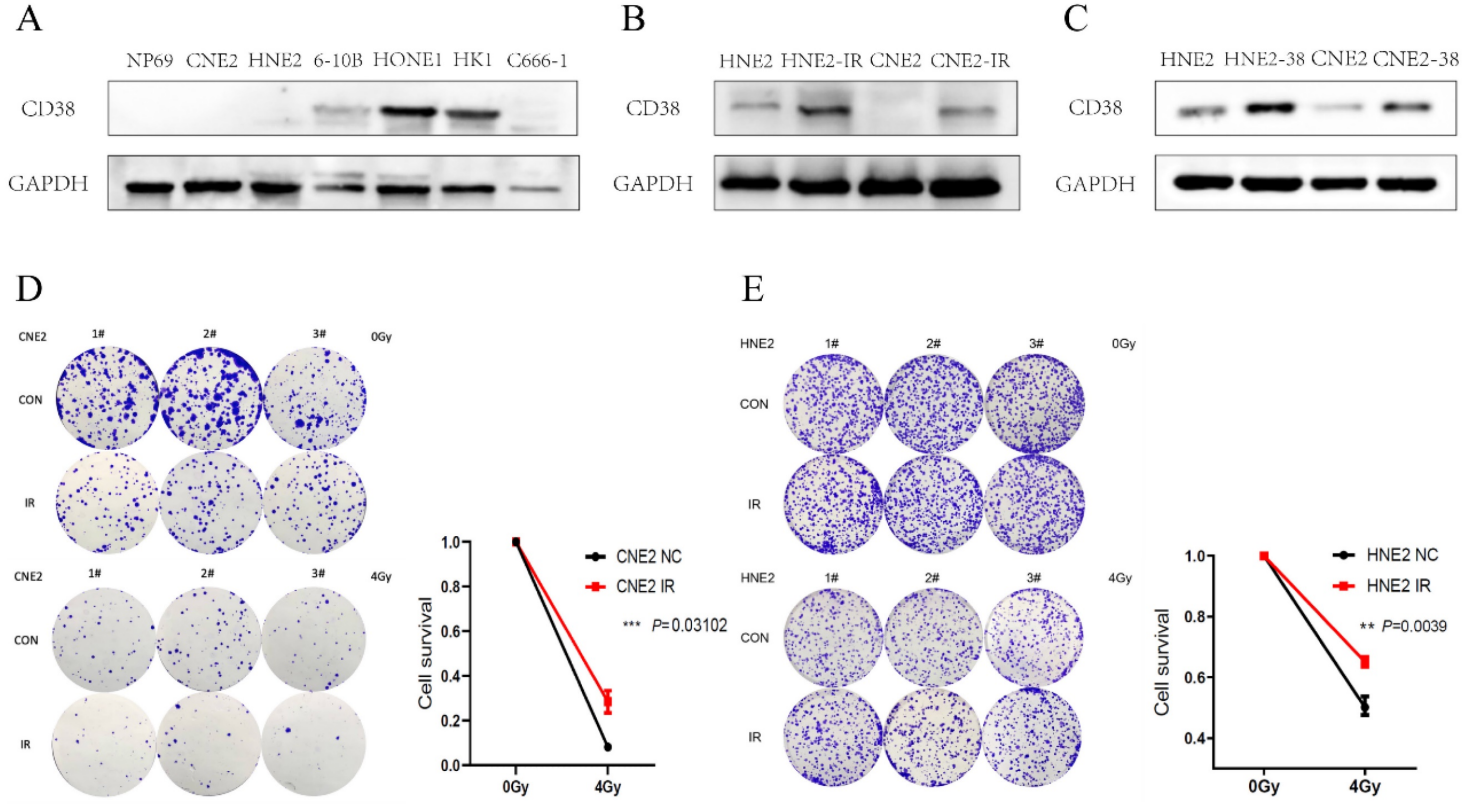
** Expression of CD38 in nasopharyngeal carcinoma cell lines. (A)** Western blotting analysis showing the levels of CD38 in nasopharyngeal carcinoma epithelial cells (NP69) and different nasopharyngeal carcinoma cell lines (CNE2, HNE2, 6-10B, HONE1, HK1, and C666-1). **(B)** Detection of CD38 levels in radiotherapy-tolerant cell lines HNE2-IR and CNE2-IR. **(C)** HNE2 and CNE2 cells were both transfected with the *CD38* expression vector and control vector, CD38 levels were detected using immunoblotting. **(D,E)** Clone formation experiment to verify the successful construction of HNE2-IR and CNE2-IR. The experiments were performed in triplicate and repeated at least two times. Student's t test was used for statistical analysis. * P < 0.01; ** P < 0.001.

**Figure 8:**
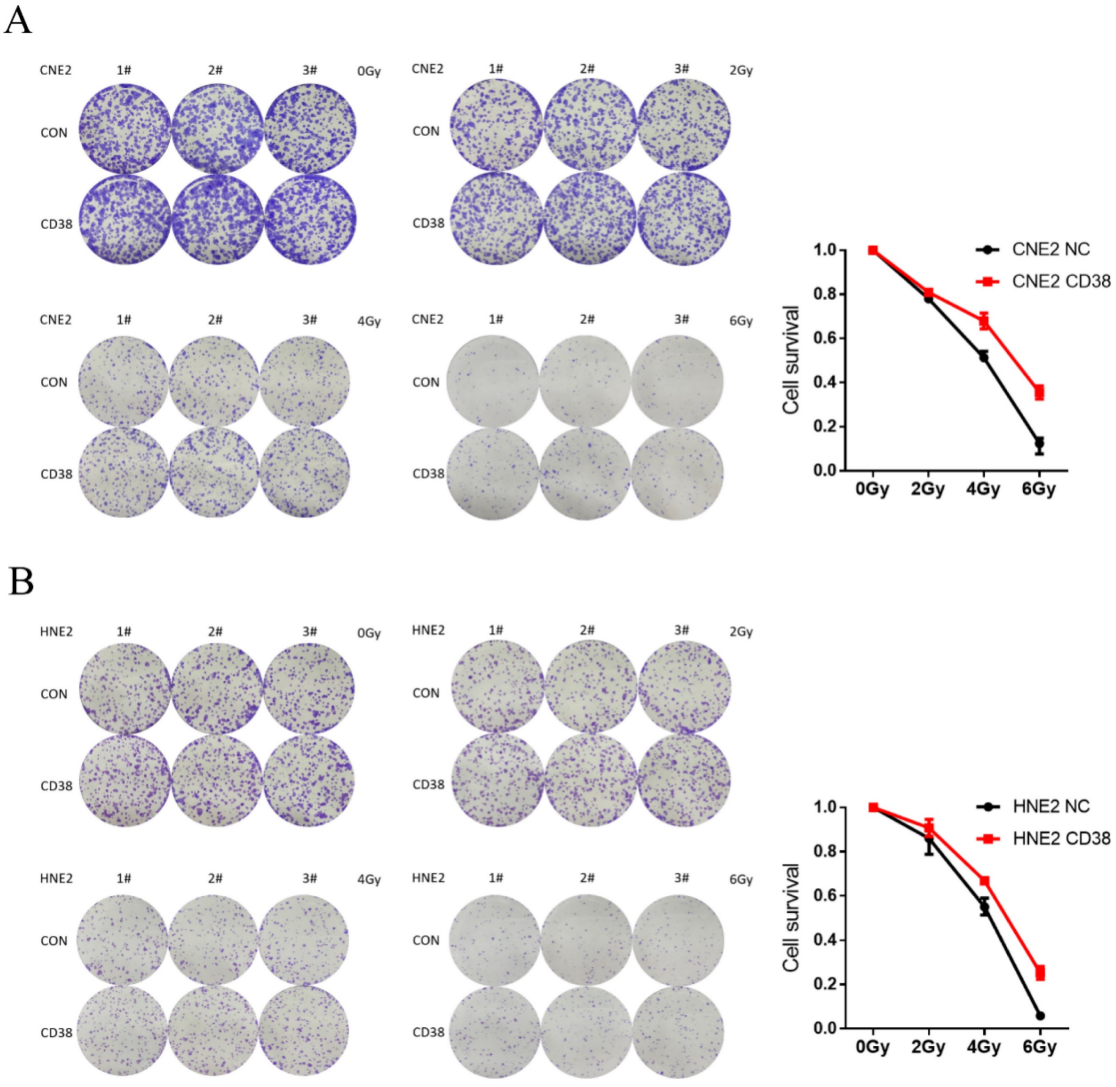
** Resistance in CD38-high cells to radiation treatment at 4 Gy and 6 Gy. (A,B)** The effects of *CD38* overexpression on colony formation in HNE2 and CNE2 cells under radiation dose of 0, 2, 4, and 6 Gy. The experiments were performed in triplicate and repeated at least three times. Student's t test was used for statistical analysis. **, P < 0.01; ***, P < 0.001.

**Figure 9:**
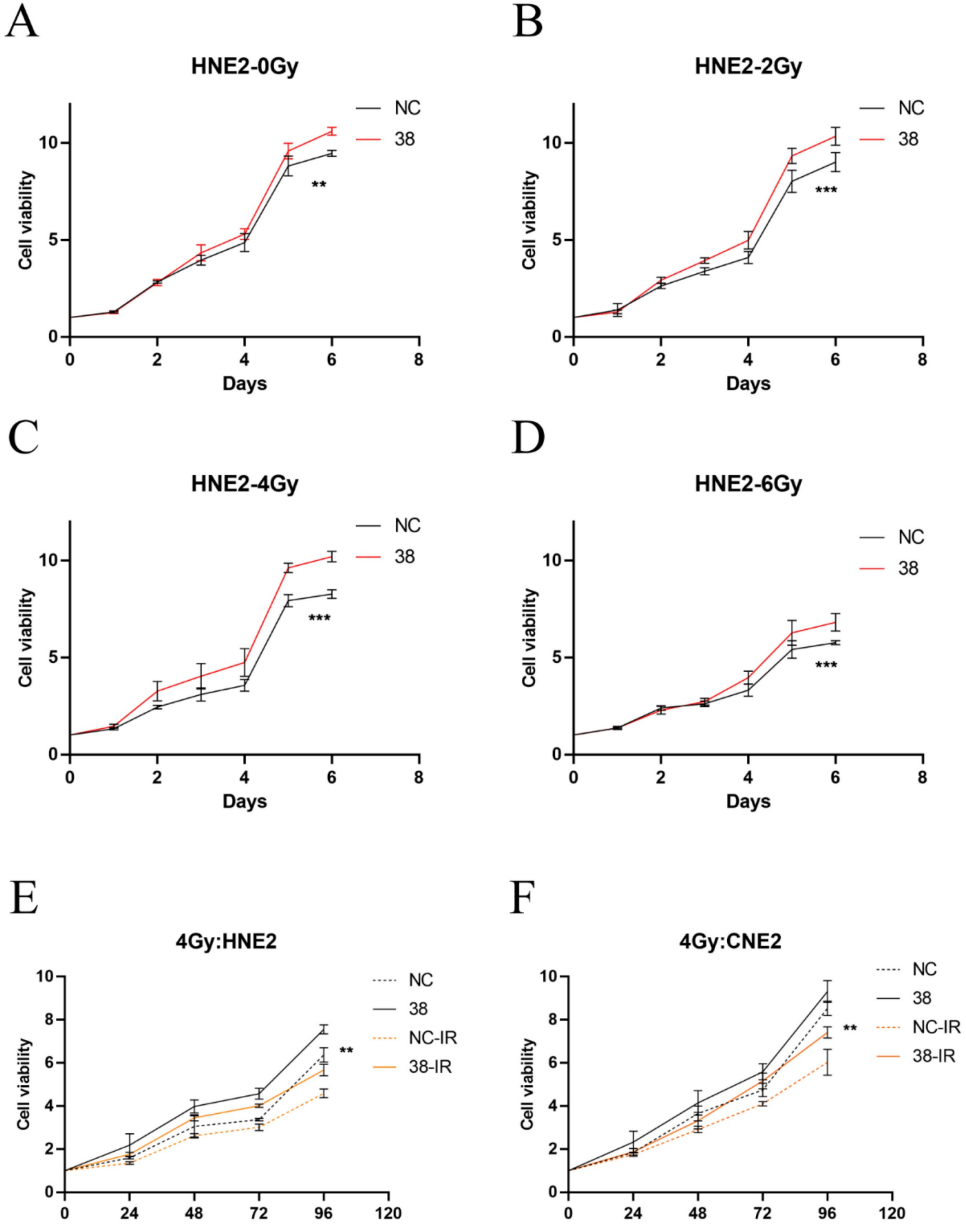
**Cell proliferation of HNE2 and CNE2 under radiation. (A-D)** CCK-8 results in HNE2 cells under radiation at 0, 2, 4, and 6 Gy. **(E,F)** Proliferation of HNE2 and CNE2 cells irradiated by 4Gy. Student's t test was used for statistical analysis. **, P < 0.01; ***, P < 0.001.

**Figure 10:**
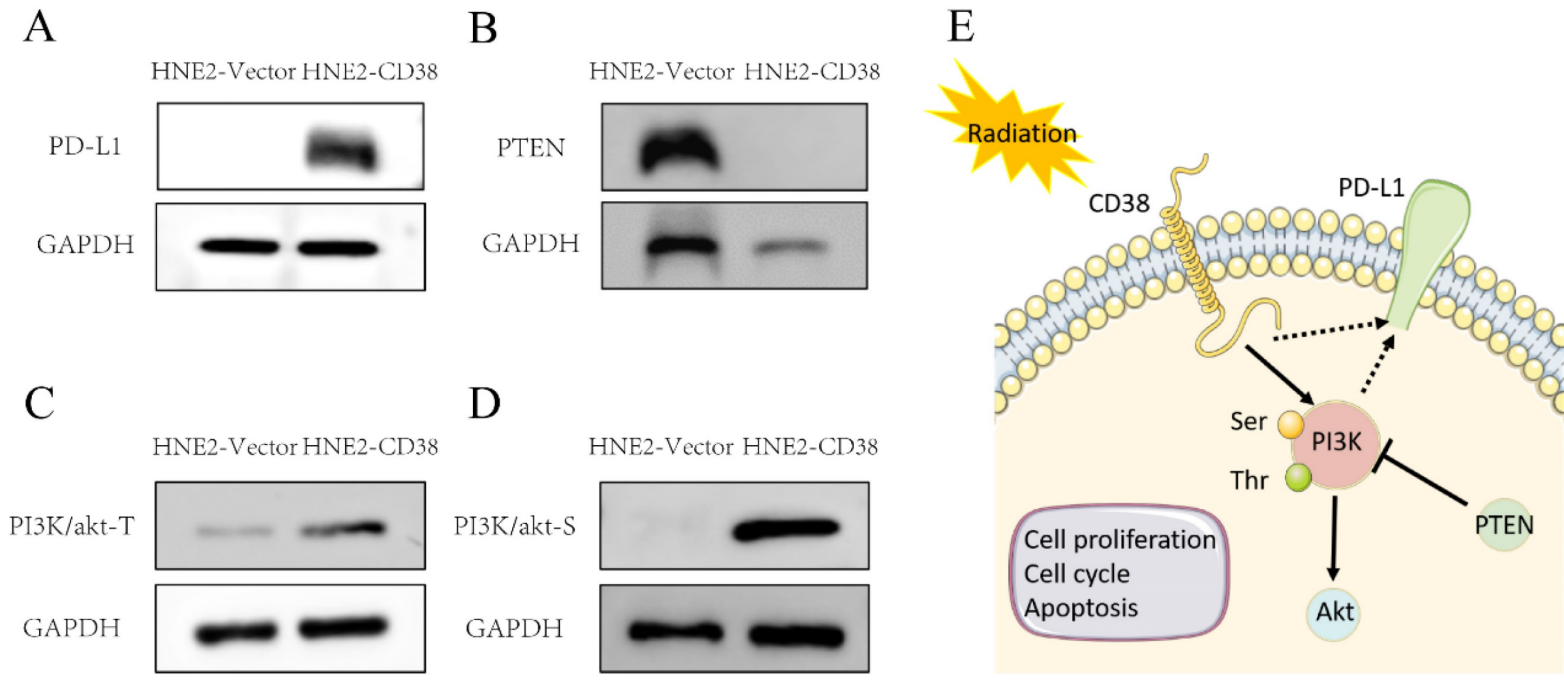
**Activation of the PI3K pathway induced by overexpression of CD38.** Western blotting showed that the level of PD-L1 **(A)** and PI3K phosphorylated at Tyr607 and Ser605 **(C,D)** and were increased, while the level of PTEN **(B)** was decreased after overexpression of CD38.

We detected the expression of CD38 (Figure 7A) in NPC cell lines, and selected CNE2 and HNE2 as experimental cell lines. Then, we overexpressed *CD38* (Figure 7C) in HNE2 and CNE2 by plasmid transfection. The effect of CD38 on the proliferation of NPC cell lines was investigated using CCK-8 and colony formation assays. After overexpression of *CD38*, the NPC cell lines showed increased proliferation and became resistant to radiotherapy (Figure 8A,B and Figure 9). To verify this observation, using weekly radiotherapy screening of NPC cells, we constructed radiotherapy-tolerant cell lines CNE2-IR and HNE2-IR (Figure 7C), and detected the levels of CD38 in the radiotherapy-tolerant cell lines and parallel parent cell lines. The results showed that the expression of CD38 increased significantly in the radiotherapy-tolerant cell lines (Figure 7B). To explain this phenomenon, we performed a preliminary study of the downstream mechanism. We found that after overexpression of CD38, the PI3K/AKT pathway was activated (Figure 10B-D), and the activation of this pathway led to the proliferation and metastasis of HNSC cells, which was consistent with the results of our previous studies and others 8, 28. Notably, after the upregulation of CD38, we found that the expression of PD‑L1 was also upregulated. Next, we stained pathological sections of NPC patients (Table S1, n=15) treated with PD-L1-targeted therapy (positive for PD-L1 as described in the medical records) with anti-CD38 antibody (Figure S8) and found positive staining in 86.7% of patients (13/15). Our results suggest that CD38 and PD-L1 are co-expressed in NPC, and some studies suggest that CD38 is positively expressed in patients with non-small cell lung cancer under the condition of PD-L1 resistance 27. In NPC patients, CD38 positivity may induce the expression of PD-L1, which will be our future research direction.

**Figure 11:**
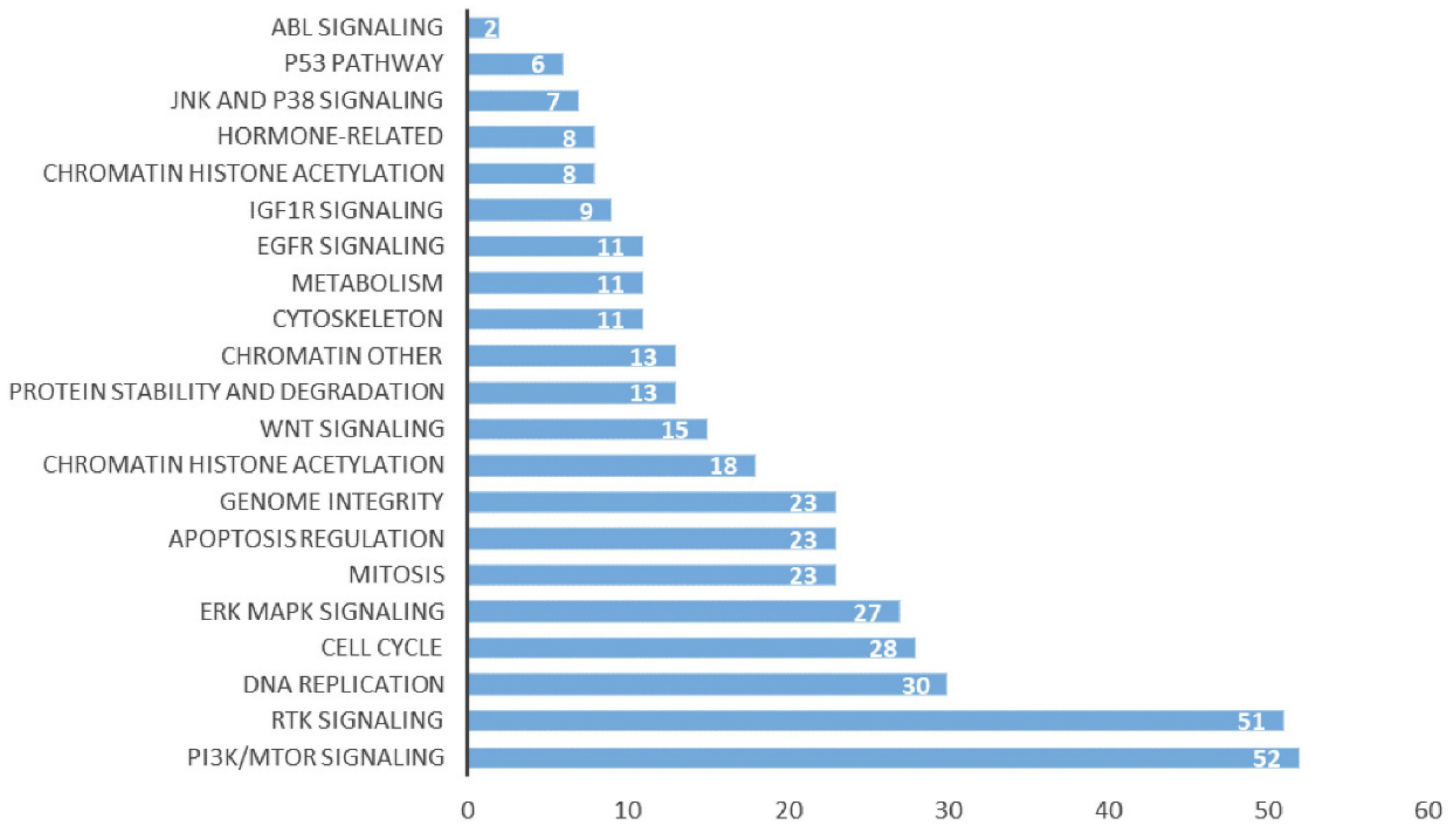
Signal pathways of tumor chemotherapy resistance.

Our previous studies showed that the high expression of CD38 promoted the transition of cells from the G2/M phase to the G0/S phase 29. In the Kaplan-Meier plotter database, we found that the high expression of CD38 in combination with chemotherapeutic drugs acting on G1/S phase suggested a poor prognosis (Figure S1), while the high expression of CD38 was associated with a better prognosis when using chemotherapeutic drugs acting on cells in other phases). Then, by performing an analysis in the GDSC database, we summarized 21 signal pathways targeted by 518 chemotherapeutic drugs used to treat tumors, as shown in Figure 11, in which the PI3K/mechanistic target or rapamycin (mTOR) pathway was the most enriched pathway. Furthermore, we detected the upregulated expression of PI3K/AKT pathway members (Figure 10 and S7) in the NPC cell lines overexpressing CD38. Notably, up-regulation of CD38 protein level was accompanied by up-regulation of NF-κb level, which may indicate that CD38 plays a key role in promoting cellular inflammatory response and immune response. At present, PI3K inhibitors have been approved for targeted therapy in the treatment of advanced breast cancer 30. We speculated that CD38 might also lead to chemotherapy tolerance by activating the PI3K-mTOR pathway 31-35. The use of PI3K inhibitors and CD38 monoclonal antibodies might improve the radiotherapy and chemotherapy tolerance, this requires further experimental research.

## Discussion

Radiotherapy is an indispensable treatment for HNSC; however, radiation resistance is the main cause of treatment failure. Therefore, the identification of drug-resistant molecules might have further clinical applications in individualized radiotherapy. To achieve this goal, we have made several important findings in this study. (1) CD38 is highly expressed in HNSC. (2) CD38 is associated with poor prognosis of HNSC. (3) The expression of CD38 is closely related to the immune infiltration of HNSC. (4) Analysis in HNSC cell lines and samples from patients with HNSC indicated that radiation resistance might be regulated through the PI3K-AKT signaling pathway. In the future, we will conduct further subgroup analyses in patients with HNSC to validate these findings, such as patients treated with combined immunotherapy, treated with chemotherapy drugs during the use of G1/S, and those with HPV infection.

NPC cells with high expression levels of CD38 showed stronger invasiveness and stronger resistance to radiation (Figure 8). Compared with untreated relatively sensitive NPC cell lines, the expression of CD38 was upregulated in radiation-tolerant cell lines. In addition, we observed that CD38 overexpression-mediated resistance made HNE2 cells more aggressive (Figure 8) when treated with ionizing radiation. In contrast, relatively low expression of CD38 reduced NPC cell growth and increased their sensitivity to radiation. In addition, upon upregulation of CD38 expression, we observed an increase in the expression of PD-L1 (Figure 7), and bioinformatic analyses also showed the aggregation of tumor infiltrating immune cells (TIICs) (Figure 5,6), which indicated that the regulation by CD38 of the immune microenvironment requires further study.

There is growing evidence that CSCs are particularly resistant to chemotherapy and radiotherapy, which might lead to treatment failure. Residual CSCs is one of the mechanisms of immune drug resistance in HNSC. Researchers have used this as a target to strengthen immunotherapy and reduce tumor recurrence and metastasis 36-40. In HNSC, stem cell markers, including CD133, CD44, and aldehyde dehydrogenase 1 family member A1 (ALDH1), can be used effectively to evaluate CSCs 41-51. Our previous results show that CD38 is a reliable CSC marker in NPC 29. Consistent with these results, we found that radiation-resistant NPC cells had higher CD38 expression, while NPC cells overexpressing CD38 showed radiation-resistant biological behavior. These data suggested that CSC characteristics might be the seeds of tumor growth, and targeting CD38 may be an effective method for targeting CSCs (Figure S2). The results of the present study suggested that targeting the CD38-PI3K/AKT axis may be valuable to treat HNSC.

Sensitivity to radiation depends on the phase of the cell cycle 52-54. Radiation‑induced damage, such as DNA single strand breaks and interchain cross‑linking, is toxic in the S phase, but relatively non-toxic in the G1 phase 55. The increase in the inherent resistance to DNA damage agents and in the DNA repair rate results from G1 phase arrest, which might enhance radiation resistance 56-57. We found that overexpression of CD38 could induce G1 phase arrest in NPC cells. However, there is a lack of prognosis data related to patients with HNC under treatment with chemotherapy drugs and CD38 expression; meanwhile, we found it very interesting that patients with higher expression of CD38 suggest a lower prognosis, for example ovarian cancer patients were treated with chemotherapy drugs that specific affect cells in the G1/S phase (Figure S1), as our previous experimental results suggested 29. Therefore, CD38 might promote the transformation of cells to G1/S phase, which leads to chemotherapy resistance.

Finally, the PI3K-AKT pathway plays an important role in regulating various cellular functions, including metabolism, growth, proliferation, survival, transcription, and protein synthesis, which are also closely related to radiation resistance 58-63. PI3K-AKT pathway inhibitors combined with immunotherapy are also being studied 64. By acting on the TSC complex subunit 1 (TSC1)/TSC2 complex and the mechanistic target of rapamycin complex (mTOR) signal transduction, AKT might mediate a variety of cellular functions, including cell proliferation, differentiation, invasion, tumor angiogenesis, and metastasis 65-68. In this study, we further noted that the upregulation of CD38 plays an important role in promoting the radiation resistance of HNSC (Figure 7). Our findings were consistent with the results of analysis in different databases, that is, the expression of CD38 in HNSC is higher than that in the normal head and neck squamous epithelium, which may be related to radiation resistance and the combined use of chemotherapy drugs against cells in the G1/S phase. We also proved that CD38 is closely related to poor prognosis of patients with HNSC receiving radiotherapy (Figure 4). These results are also supported by other studies. CD38 is overexpressed in several types of cancer, including prostate cancer, lung cancer, colorectal cancer, cervical cancer, and nasopharyngeal carcinoma, in which it is associated with poor prognosis 29, 69-74. In addition, the use of PI3K-AKT inhibitors might increase radiosensitivity by regulating their molecular expression as a therapeutic target 75-77. All these results highlight the importance of the CD38-PI3K/AKT axis in radiation resistance and cancer invasiveness. CD38 inhibitors have been shown to be effective against melanoma in different mouse models 27. In this study, we found that upregulation of CD38 activated the PI3K-AKT pathway; therefore, we believe that inhibition of both CD38 and PI3K might improve the prognosis of patients with HNSC.

In a previous study, we reported that CD38 is highly associated with late staging and poor prognosis of nasopharyngeal carcinoma 8-9. In this study, we studied the expression of CD38 in HNSC in different databases and the changes of biological behavior of NPC cells after regulating the expression of CD38. In our previously reported cervical cancer study 29, high expression of CD38 could induce cell cycle changes in cervical cancer cells and promote cell cycle arrest in the G1/S phase. Interestingly, we found that the expression of PD-L1 was also increased in NPC cell lines with high expression of CD38. Recently, Anderson reported the role of CD38 in immune drug resistance of PD-1 27. Another report showed that the resistance of hepatocellular carcinoma to PD-1/PD-L1 antibodies might be caused by the upregulation of CD38 in tumor cells 78. Our results also support the argument that CD38 targeted therapy in combination with PD-1 targeted drugs might be a promising strategy in patients with HNSC to achieve better OS. This concept is consistent with current clinical methods, because in most cases, the value of a single drug is limited. Cancer cells are frequently reprogrammed, and multi‑target/multi‑pathway inhibition might be a better strategy to treat tumors. However, the safety of combination therapy remains a concern 79-83. Although there have been many recent reports the lack of response to immunotherapy or early recurrence 84, we are encouraged by the results of PD-1/PD-L1 sensitization in HNSC radiotherapy 85. In summary, our results suggested that activation of the PI3K-AKT pathway in nasopharyngeal carcinoma cells might be the driver of acquired radiation resistance, which prompted us to further explore the relationship of the PI3K-AKT pathway in nasopharyngeal carcinoma cells, such as whether the phenotypic changes are caused by downstream mechanistic changes through protein-protein interactions (Figure S6), which will be further studied in the future.

A comprehensive bioinformatic analysis showed that CD38 was highly expressed in a variety of HNSC samples (Figure S3-5) and was associated with poor prognosis; at the same time, the expression of CD38 correlated highly with a variety of immune infiltrating cells. Here, we proved that CD38 is overexpressed in radiation-tolerant NPC cell lines, and the overexpression of CD38 in NPC cell lines led to activation of the PI3K/AKT pathway, thus enhancing NPC cell radiation resistance. Therefore, our results provide a preliminary theoretical basis for the treatment of patients with CD38‑activated drug-resistant HNSC. As a new biomarker, CD38 will be helpful to guide the personalized treatment of patients with HNSC patients. Future studies are required to explore the mechanism of CD38 in immune tolerance and drug resistance in radiotherapy and chemotherapy.

## Supplementary Material

Supplementary figures and table.

## Figures and Tables

**Table 1:**
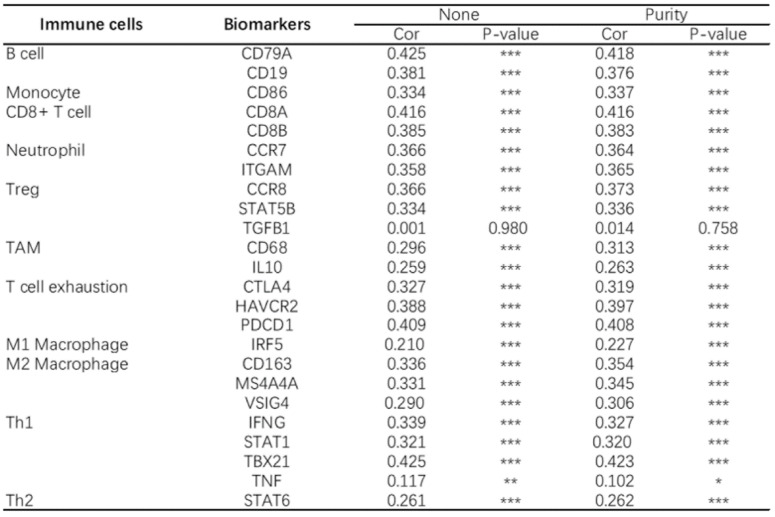
Analysis of the correlation between CD38 in HNSC and biomarkers of immune cells (TIMER)

## Abbreviations

CD38: cluster of differentiation 38
PI3K: phosphatidylinositol 3-kinase
Akt (PKB): protein kinase B
PTEN: phosphatase and tensin homolog deleted on chromosome ten
PD-1/PD-L1: programmed cell death 1/programmed cell death ligand 1
