# Supplementary material for: Integrative Analysis Identified CD38 As a Key Node That Correlates Highly with Immunophenotype, Chemoradiotherapy Resistance, And Prognosis of Head and Neck Cancer
Source: J Cancer. 2023 Jan 1;14(1):72–87. doi: 10.7150/jca.59730 (PMC9809333; doi:10.7150/jca.59730)
Supplement: Supplementary file 1 — Supplementary figures and table. [file jcav14p0072s1.pdf]

Supplementary figure legends

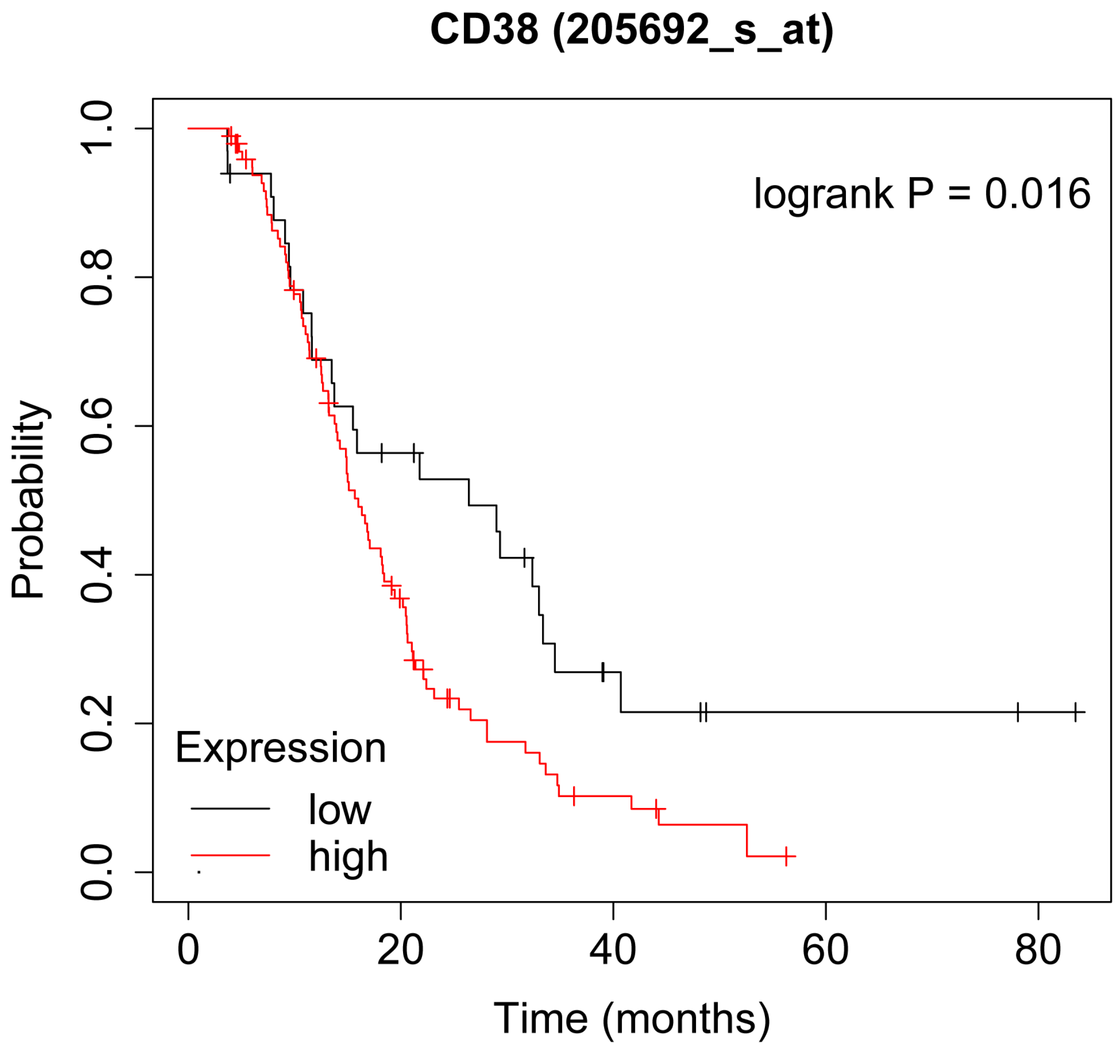

Figure S1. High expression of CD38 in the Kaplan–Meier database indicates poor prognosis in patients with ovarian cancer treated with gemcitabine (P = 0.016).

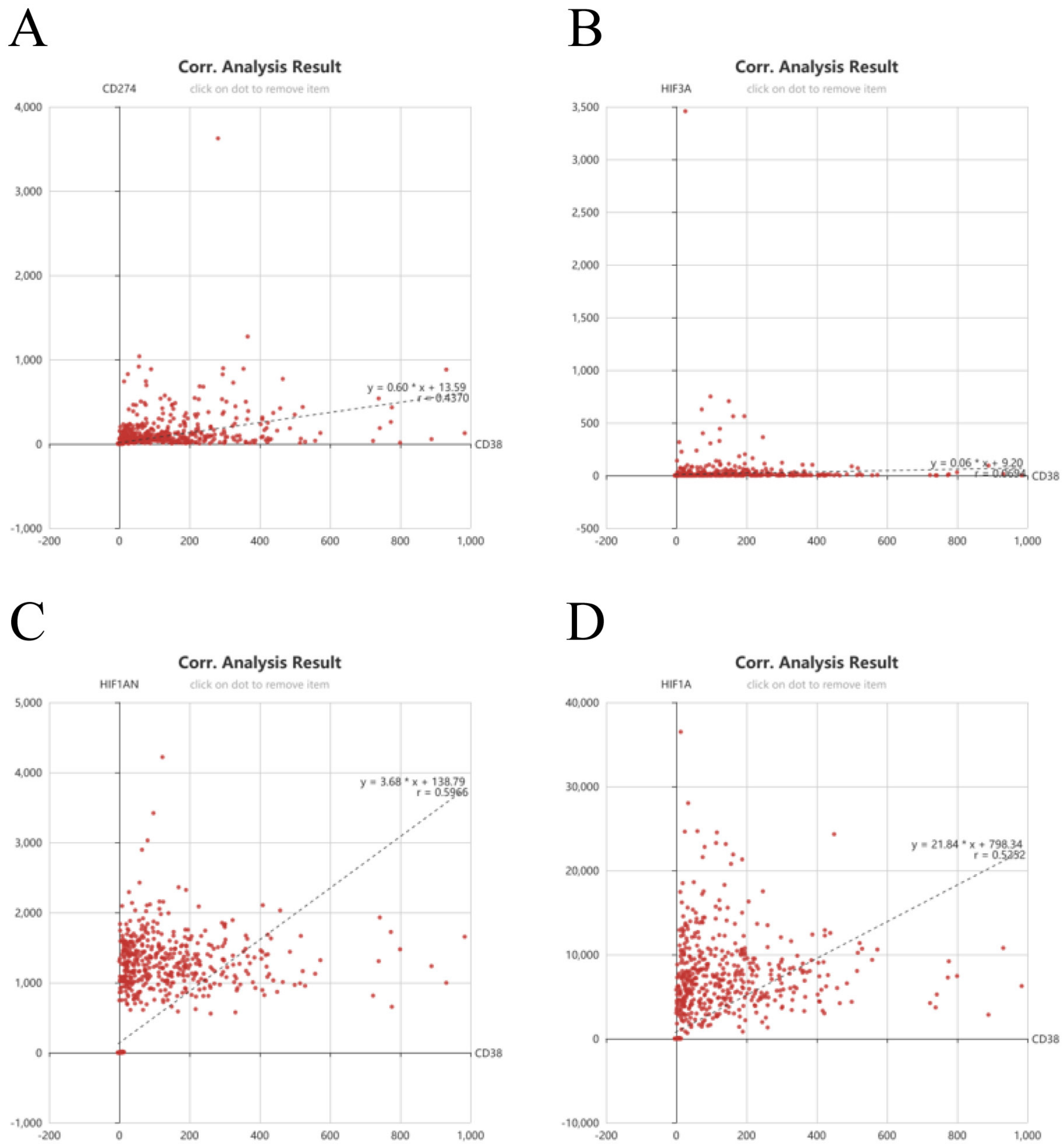

Figure S2. CD38 is closely related to PD-L1 (CD274) and the hypoxia inducible factor family (HNC Database).

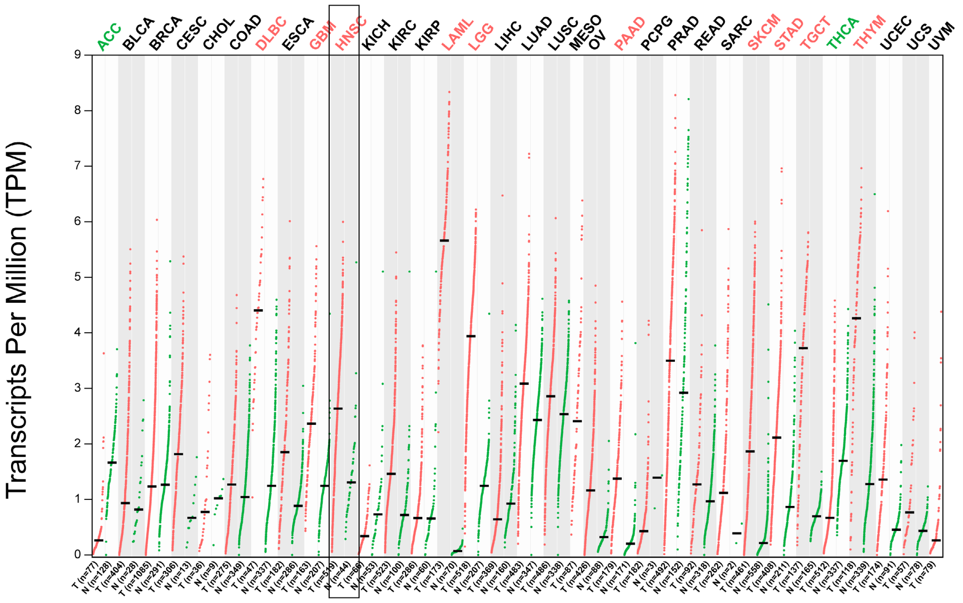

Figure S3. *CD38* expression in different types of human cancers (GEPIA). The expression level of *CD38* across 33 TCGA tumors compared with that in TCGA normal and GTEx data, shown as a scatter diagram. Red shows each TCGA tumor, and its corresponding normal and GTEx data is green. Y-axis: transcripts per million (log2 (TPM+1)); X-axis: tumor and normal tissues number. T: tumor; N: normal.

A B C

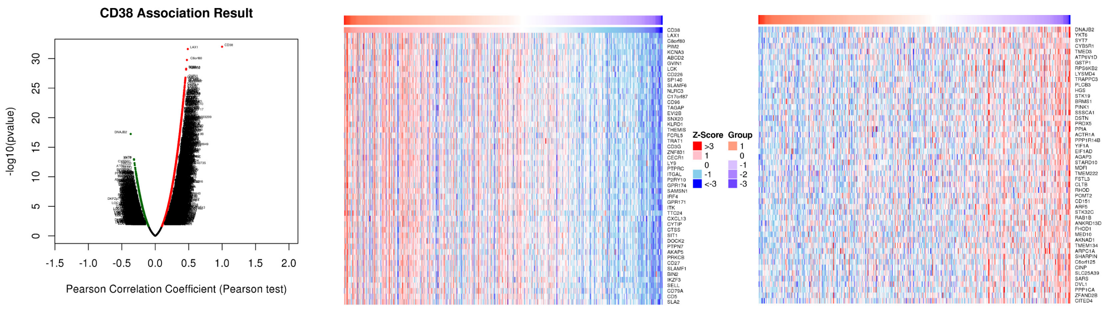

Figure S4. Enrichment analysis of CD38 in head and neck squamous cell carcinoma (LinkedOmics). (A-C) GO analysis of CD38 co-expressed genes in HNSC, including biological process, cellular component, and molecule function. (D) KEGG pathway analysis of CD38 co-expressed genes in HNSC.

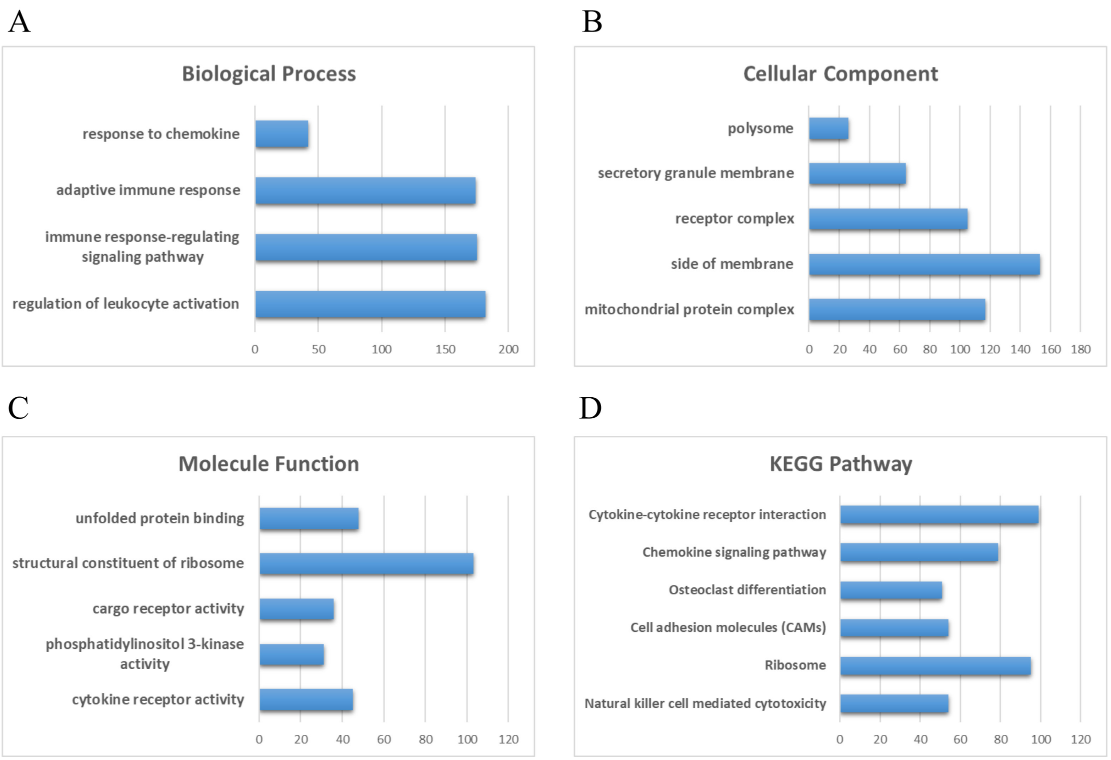

Figure S5. Differentially expressed genes that correlate with CD38 expression in head and neck squamous cell carcinoma (LinkedOmics). (A) Volcano plot showing that differentially expressed genes that correlated with CD38 in HNSC using Pearson's test. (B-C) Heat maps showing that the top 50 genes that correlated with CD38 in HNSC. Red demonstrates positively correlated genes and green demonstrates negatively correlated genes.

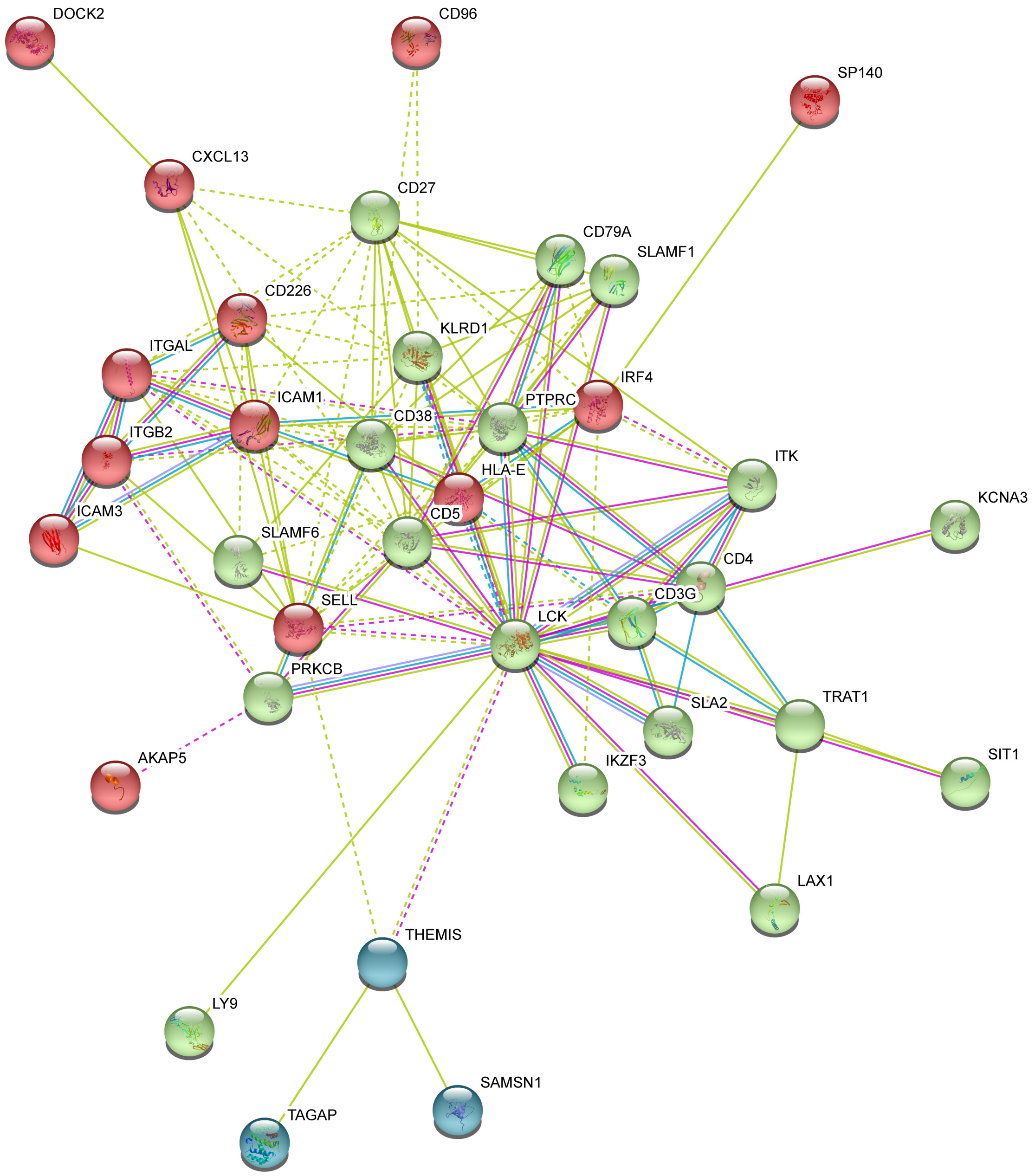

Figure S6. Cluster Analysis of the Protein-Protein Interaction Networks (PPI) of CD38 in the Human body (STRING).

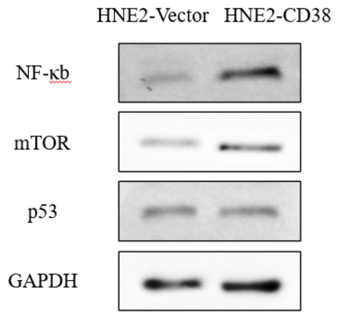

Figure S7. Activation of the PI3K pathway.

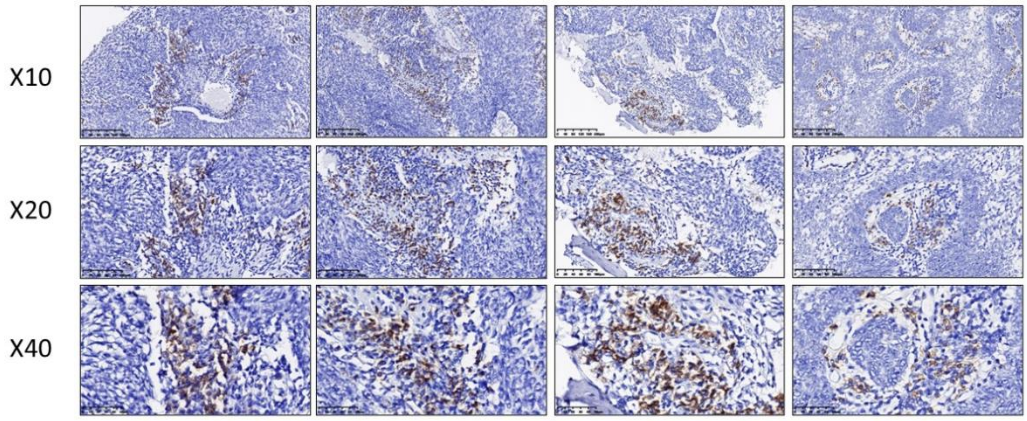

Figure S8. CD38 expression in NPC patients.

Parameters	Number
Sex	
Male	12
Female	3
Age (years)	

<60	4
≥60	8
EBV	
Positive	9
Negative	6
TNM Stage	
I, II	4
III, IV	11
TNM, Tumor, node and metastasis.	

Table S1. Characteristics of NPC patients.
